# Rectal washout does not increase the complication risk after anterior resection for rectal cancer

**DOI:** 10.1186/s12957-021-02193-7

**Published:** 2021-03-19

**Authors:** Karl Teurneau-Hermansson, Rebecca Svensson Neufert, Pamela Buchwald, Fredrik Jörgren

**Affiliations:** 1 Department of Surgery, Helsingborg Hospital, Helsingborg, Lund University, Lund, Sweden; 2 Department of Surgery, Skåne University Hospital, Malmö, Lund University, Lund, Sweden

**Keywords:** Rectal, Cancer, Washout, Complications, Resection

## Abstract

**Background:**

To reduce local recurrence risk, rectal washout (RW) is integrated in the total mesorectal excision (TME) technique when performing anterior resection (AR) for rectal cancer. Although RW is considered a safe practice, data on the complication risk are scarce. Our aim was to examine the association between RW and 30-day postoperative complications after AR for rectal cancer.

**Methods:**

Patients from the Swedish Colorectal Cancer Registry who underwent AR between 2007 and 2013 were analysed using multivariable methods.

**Results:**

A total of 4821 patients were included (4317 RW, 504 no RW). The RW group had lower rates of overall complications (1578/4317 (37%) vs. 208/504 (41%), *p* = 0.039), surgical complications (879/4317 (20%) vs. 140/504 (28%), *p* < 0.001) and 30-day mortality (50/4317 (1.2%) vs. 12/504 (2.4%), *p* = 0.020). In multivariable analysis, RW was a risk factor neither for overall complications (OR 0.73, 95% CI 0.60–0.90, *p* = 0.002) nor for surgical complications (OR 0.62, 95% CI 0.50–0.78, *p* < 0.001).

**Conclusions:**

RW is a safe technique that does not increase the 30-day postoperative complication risk after AR with TME technique for rectal cancer.

## Background

Due to advances in management of rectal cancer during the last decades, oncological outcome has improved in terms of decreased local recurrence (LR) rate and improved survival [1–8]. Mainly, these improvements are due to the introduction of the total mesorectal excision (TME) technique and preoperative radiotherapy (RT) or chemoradiotherapy in selected cases [4, 6, 8]. Among resected rectal cancer patients in Sweden, the 5-year LR rate is < 5% [9].

A potential source for LR in rectal cancer is implantation of intraluminal, viable malignant cells shed from the tumour [10–14]. To eliminate those cells and thereby reduce the LR risk, rectal washout (RW) has been an integrated part of the TME technique when performing anterior resection (AR) for rectal cancer. The impact of RW on the LR rate is controversial [15–23]. RW is stated to be inexpensive, easy and quick to perform as well as a safe procedure [15, 16, 19, 23]. However, regarding the safety of RW, the data are scarce. There are no clear contraindications of RW. A few case reports describing procedure-related complications have been published involving blood pressure drop and cardiac ischemia after RW with cetrimide and anaphylaxis after RW with chlorhexidine [24, 25]. In addition, during minimally invasive surgery, RW can sometimes be difficult and time-consuming due to that the proximal clamping is technically challenging [26].

Postoperative complications following rectal cancer surgery are common. Population-based registries report rates between 30 and 40% [9, 27–29]. To our knowledge, no study has explored a potential association between RW and complication risk. We aimed to evaluate the safety of RW, using population-based data from the Swedish Colorectal Cancer Registry (SCRCR), and explore the hypothesis that RW does not increase the 30-day postoperative complication risk with a focus on surgical complications.

## Methods

Since 1995, all diagnosed rectal cancers in Sweden have been prospectively registered in the SCRCR [5, 9]. Data related to the patient, the tumour, the preoperative assessment, the treatment and postoperative complications are registered 30 days after surgery or at diagnosis for patients not treated with surgery. Follow-up data with information about adjuvant treatment, late postoperative complications, recurrences and death are registered after three and five years. The SCRCR covers approximately 99% of all patients diagnosed with rectal cancer in Sweden [5, 9]. The internal data validity has proven to be high, and the SCRCR has been described in detail in other publications [5, 28, 30].

This study was approved by the Ethical Review Board of Lund University, Sweden (Dnr2014/332), and followed the Declaration of Helsinki guidelines. We retrieved data on all patients with rectal cancer registered in the SCRCR between 2007 and 2013. Patients subjected to AR who had available data on RW were selected for further analysis. The study cohort was subdivided into two groups—RW and no RW. Differences in patient and tumour characteristics as well as treatment and early postoperative complications were calculated. The following complications were included: 30-day mortality, reoperations, and infectious, cardiovascular, neurological and surgical complications. Surgical complications were subdivided into wound infections, intraabdominal infections, wound dehiscences, intraabdominal bleedings, anastomotic leakages (AL) and stoma complications.

Rectal cancer is defined as an adenocarcinoma that is completely or partly located within 15 cm from the anal verge measured with rigid sigmoidoscopy during withdrawal. RW denotes intraoperative irrigation of the rectum after cross-clamping below the tumour but above the intended anastomosis line before transection, to eliminate exfoliated malignant cells. The SCRCR includes whether RW was performed or not but does not describe solution, volume or technique used. Early postoperative complications are defined as complications occurring within 30 days of surgery both in hospital and after discharge. TME is defined as sharp dissection under direct vision in embryological avascular planes with removal of the rectum including intact mesorectum down to the pelvic floor. For most of the highly situated tumours, partial mesorectal excision was performed (i.e., division of the rectum and the mesorectum 5 cm below the tumour). A hospital that annually performs > 25 major abdominal procedures for rectal cancer is defined as a high-volume hospital. A colorectal surgeon is an accredited colorectal surgeon or a surgeon with special interest in colorectal surgery trained in the TME technique. A locally radical procedure (R0) is defined as no macroscopic tumour growth left after completed surgery as judged by the surgeon and no microscopic tumour growth at the resected specimen margins as judged by the pathologist (circumferential resection margin (CRM) > 1 mm). When there is disagreement, the resection is classified as an R1-procedure (also including the group of patients with CRM ≤ 1 mm). If both the surgeon and the pathologist agree that tumour growth is left behind, the resection is by definition a R2-procedure.

### Statistical analysis

Categorical variables were presented as number and proportions in percentages. Numerical data were reported as median with interquartile range. Chi-square test, Fisher’s exact test and two sample *T*-tests were used for intergroup comparisons when appropriate. When calculating differences between groups, missing data were excluded. For complications with a sufficient number of events, univariable analysis was performed on potential risk factors (i.e., age, gender, ASA-class, BMI, low-/high-volume hospital, tumour height, RT, TNM stage, temporary stoma, perforation, residual tumour status, colorectal surgeon and laparoscopic procedure). Univariable analysis of early postoperative complications was performed, and relevant variables, as specified in the “Results” section, were included in multivariable analysis. Logistic regression was used to analyse RW effect on complications, both univariable and multivariable adjusted for clinically important confounding variables. Due to event distribution with all events occurring in either group, residual tumour status and colorectal surgeon were not adjusted when analysing 30-day mortality, and residual tumour status was not adjusted when analysing cardiovascular complications. For all tests, *p* values < 0.05 were considered statistically significant. All statistical analyses were conducted using IBM® SPSS® Statistics version 23.00 for Windows® (IBM Corp, Armonk, NY, USA).

## Results

**Fig. 1:**
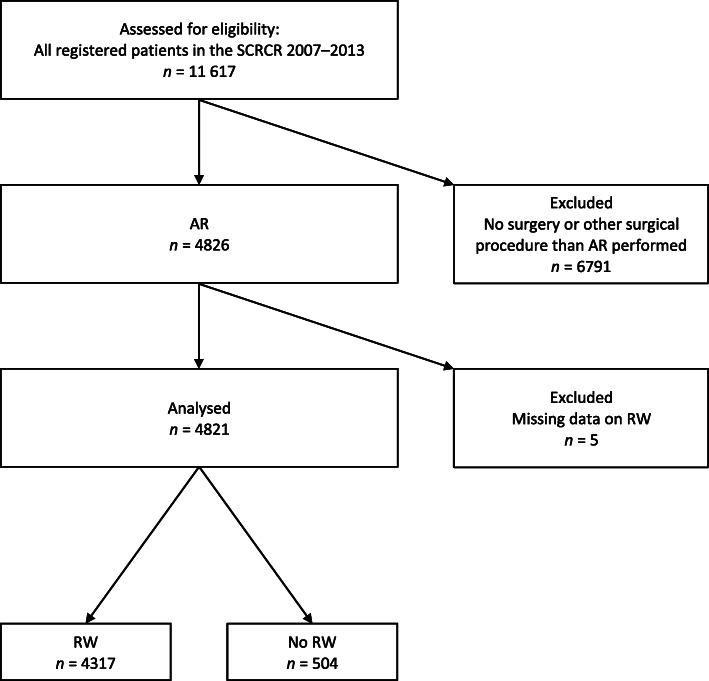
Flow chart showing the selection of patients for inclusion in the study. SCRCR, Swedish Colorectal Cancer Registry; AR, anterior resection; RW, rectal washout

Between 2007 and 2013, 11,617 patients with rectal cancer were registered in the SCRCR. AR was performed in 4826/11,617 (41.5%) patients. After exclusion, 4821 patients were included for analysis (Fig. 1). RW was performed in 4317/4821 (89.5%) and not performed in 504/4821 (10.5%) of analysed patients.

Patient, tumour and treatment characteristics are shown in Table 1. In the RW group, more patients had preoperative RT (*p* < 0.001), had surgery performed by a colorectal surgeon (*p* < 0.001), and R0 resections were more prevalent (*p* < 0.001). In addition, the operation took longer (*p* < 0.001), and temporary stoma was more frequent (*p* < 0.001). In the no RW group, the tumours were higher situated (*p* < 0.001), and the patients had more advanced TNM-stages (*p* = 0.005). More emergency procedures (*p* < 0.001), laparoscopic procedures (*p* < 0.001) and intraoperative perforations (*p* < 0.001) occurred in the no RW group. Other studied variables were equally distributed between the groups.

**Table 1:** Demographic data of patients registered in the SCRCR 2007–2013 undergoing anterior resection for rectal cancer presented as all patients, rectal washout and no rectal washout

		All patients (*n* = 4821), *n* (%)	RW (*n* = 4317), *n* (%)	No RW (*n* = 504), *n* (%)	*p* value
Age at primary surgery (years)^a^		67 (14)	67 (14)	68 (16)	0.057
Gender	M	2839 (58.9)	2543 (58.9)	296 (58.7)	0.939
	F	1982 (41.1)	1774 (41.1)	208 (41.3)	0.939
ASA score	I	1183 (24.5)	1048 (24.3)	135 (26.8)	0.373
	II	2773 (57.5)	2504 (58.0)	269 (53.4)	
	III	739 (15.3)	658 (15.2)	81 (16.1)	
	IV	31 (0.6)	28 (0.6)	3 (0.6)	
	V	0 (0)	0 (0)	0 (0)	
	Missing data	95 (2.0)	79 (1.8)	16 (3.2)	
BMI (kg/m^2^)^a^		25.31 (5)	25.31 (5)	25.15 (5)	0.985
	Missing data	361	303	58	
Tumour height (cm)	Low 0–5	155 (3.2)	135 (3.1)	20 (4.0)	<0.001
	Medium 6–10	2346 (48.7)	2149 (49.8)	197 (39.1)	
	High 11–15	2281 (47.3)	2011 (46.6)	270 (53.6)	
	Missing data	39 (0.8)	22 (0.5)	17 (3.4)	
TNM stage	I	1276 (26.5)	1138 (26.4)	138 (27.4)	0.005
	II	1388 (28.8)	1276 (29.6)	112 (22.2)	
	III	1676 (34.8)	1497 (34.7)	179 (35.5)	
	IV	378 (7.8)	326 (7.6)	52 (10.3)	
	Missing data	103 (2.1)	80 (1.9)	23 (4.6)	
Preoperative RT		2899 (60.1)	2665 (61.7)	234 (46.4)	<0.001
	Missing data	2 (0)	2 (0)	0 (0)	
Preoperative CHT		693 (14.4)	629 (14.6)	64 (12.7)	0.255
	Missing data	2 (0)	2 (0)	0 (0)	
Intraoperative perforation	No	4676 (97.0)	4202 (97.3)	474 (94.0)	<0.001
	Yes	119 (2.5)	94 (2.2)	25 (5.0)	
	Missing data	26 (0.5)	21 (0.5)	5 (1.0)	
Emergency procedure	No	4767 (98.9)	4278 (99.1)	489 (97.0)	<0.001
	Yes	29 (0.6)	18 (0.4)	11 (2.2)	
	Missing data	25 (0.5)	21 (0.5)	4 (0.8)	
Laparoscopic surgery	Yes	397 (8.2)	314 (7.3)	83 (16.5)	<0.001
	No	4392 (91.1)	3977 (92.1)	415 (82.3)	
	Missing data	32 (0.7)	26 (0.6)	6 (1.2)	
Operative time (min)^a^		231 (125)	235 (126)	207 (122)	<0.001
	Missing data	142	117	25	
Blood loss (ml)^a^		400 (500)	400 (500)	350 (500)	0.430
	Missing data	186	153	33	
Temporary stoma		3595 (74.6)	3329 (77.1)	266 (52.8)	<0.001
	Missing data	11 (0.2)	8 (0.2)	3 (0.6)	
Surgical competence	Colorectal	4728 (98.1)	4246 (98.4)	482 (95.6)	<0.001
	General	47 (1.0)	32 (0.7)	15 (3.0)	
	Missing data	46 (1.0)	39 (0.9)	7 (1.4)	
Length of hospital stay (days)^a^		10 (7)	10 (7)	10 (8)	0.220
	Missing data	13	11	2	
Radicality	R0	4116 (98.0)	3726 (98.2)	390 (95.8)	<0.001
	R1	79 (1.9)	66 (1.7)	13 (3.2)	
	R2	5 (0.1)	1 (0)	4 (1.0)	

^a^Values in parentheses are interquartile range

*RW* rectal washout, *RT* radiotherapy, *CHT* chemotherapy

Data on 30-day postoperative complications are shown in Table 2. Postoperative complications overall occurred in 1786/4821 (37.0%) patients. The overall complication rate was lower in the RW group compared to the no RW group (1578/4317 (36.6%) vs. 208/504 (41.3%), *p* = 0.039). In the RW group, 30-day mortality was lower (50/4317 (1.2%) vs. 12/504 (2.4%) respectively, *p* = 0.020) and the reoperation rate was lower (419/4317 (9.7%) vs. 76/504 (15.1%), *p* < 0.001). There were less surgical complications overall in the RW group (879/4317 (20.4%) vs. 140/504 (27.8%), *p* < 0.001) with lower rates for wound dehiscences (*p* = 0.041), AL (*p* = 0.003) and stoma complications (*p* = 0.010).

**Table 2:** Data on 30-day postoperative complications after anterior resection for rectal cancer presented as all patients, rectal washout and no rectal washout

Complications	All patients (*n* = 4821), *n* (%)	RW (*n* = 4317), *n* (%)	No RW (*n* = 504), *n* (%)	*p* value
Overall	1786 (37.0)	1578 (36.6)	208 (41.3)	0.039
Missing data	3 (0)	3 (0)	0 (0)	
30-day mortality	62 (1.3)	50 (1.2)	12 (2.4)	0.020
Missing data	6 (0.1)	3 (0.1)	3 (0.6)	
Reoperation	495 (10.3)	419 (9.7)	76 (15.1)	<0.001
Missing data	22 (0.5)	17 (0.4)	5 (1.0)	
Infectious^a^	287 (6.0)	256 (5.9)	31 (6.2)	0.843
Cardiovascular^a^	159 (3.3)	139 (3.2)	20 (4.0)	0.373
Neurological^a^	11 (0.2)	10 (0.2)	1 (0.2)	1.000
Other^a^	537 (11.1)	488 (11.3)	49 (9.7)	0.285
Surgical^a^	1019 (21.1)	879 (20.4)	140 (27.8)	<0.001
Wound infection^a^	211 (4.4)	182 (4.2)	29 (5.8)	0.110
Intraabdominal infection^a^	171 (3.5)	152 (3.5)	19 (3.8)	0.775
Wound dehiscence^a^	88 (1.8)	73 (1.7)	15 (3.0)	0.041
Intraabdominal bleeding^a^	43 (0.9)	36 (0.8)	7 (1.4)	0.210
Anastomotic leakage^a^	405 (8.4)	345 (8.0)	60 (11.9)	0.003
Stoma complication^a^	89 (1.8)	74 (1.7)	15 (3.0)	0.010
Other surgical^a^	54 (1.1)	46 (1.1)	8 (1.6)	0.292

^a^No missing data

*RW* rectal washout

Data for univariable and multivariable analyses are presented in Table 3. In multivariable analysis regarding postoperative complications overall, the odds ratio (OR) was 0.73 (95% confidence interval (CI) 0.60–0.90, *p* = 0.002), favouring the RW group. Furthermore, the RW group had advantageous outcomes regarding surgical complications with an OR of 0.62 (95% CI 0.50–0.78, *p* < 0.001), AL with an OR of 0.59 (95% CI 0.43–0.80, *p* = 0.001), and in reoperations the OR was 0.61 (95% CI 0.46–0.81, *p* = 0.001). No differences between the groups were found considering the impact of RW on other analysed postoperative complications.

**Table 3:** Univariable and multivariable logistic regression analysis of the impact of rectal washout on early postoperative complications after anterior resection for rectal cancer

Complications	Univariable odds ratio (CI)	*p* value	Multivariable odds ratio (CI)	*p* value
Overall	0.82 (0.68–0.99)	0.039	0.73 (0.60–0.90)	0.002
30-day mortality^a^	0.48 (0.25–0.90)	0.023	0.55 (0.27–1.13)	0.105
Surgical	0.67 (0.54–0.82)	< 0.001	0.62 (0.50–0.78)	< 0.001
Anastomotic leakage	0.64 (0.48–0.86)	0.003	0.59 (0.43–0.80)	0.001
Reoperation	0.60 (0.46–0.78)	< 0.001	0.61 (0.46–0.81)	0.001
Cardiovascular^b^	0.81 (0.50–1.30)	0.374	0.79 (0.47–1.33)	0.378
Infectious	0.96 (0.66–1.41)	0.843	0.92 (0.61–1.39)	0.688
Other	1.18 (0.87–1.61)	0.286	0.98 (0.71–1.37)	0.911

Multivariable analysis adjusted for age, gender, ASA-class, BMI, low-/high-volume hospital, tumour height, radiotherapy, temporary stoma, perforation, TNM stage, residual tumour, colorectal surgeon and laparoscopic procedure unless indicated otherwise

^a^Adjusted for age, gender, ASA-class, BMI, low-/high-volume hospital, tumour height, radiotherapy, temporary stoma, perforation, TNM stage and laparoscopic procedure

^b^Adjusted for age, gender, ASA-class, BMI, low-/high-volume hospital, tumour height, radiotherapy, temporary stoma, perforation, TNM stage, colorectal surgeon and laparoscopic procedure

*CI* 95% confidence interval

## Discussion

In this study, RW was not associated with early postoperative complications, neither overall nor surgical. On the contrary, complications were less frequent in the RW group compared to the no RW group. To our knowledge, this is the first study with special emphasis on this issue.

There were significant differences between the RW and no RW groups. Some differences can be attributed to the surgical competence, and there is a risk that RW acts as a surrogate marker for overall surgical quality. The proportion of procedures performed by colorectal surgeons was higher in the RW group, which might explain the lower frequency of intraoperative perforations and non-radical surgery in this group. Preoperative RT was less frequently used in the no RW group, which might be related to surgical competence and availability to multidisciplinary teams. If the operating surgeon follows one recommendation of good surgical practice, perhaps the surgeon is more prone to adhere to guidelines. Not only surgical competence but also adverse intraoperative events and the higher proportion of emergency procedures in the no RW group may be contributing factors. Data on bowel preparation and perioperative antibiotics were not available for this study. However, the national guidelines recommend prophylactic antibiotics and preoperative bowel preparation in AR [31]. To reconcile differences between the RW and no RW groups, we adjusted for possible confounders using multivariable analyses. Despite this, reduced postoperative complications following RW were found.

The main argument for performing RW is the reduced LR risk. The evidence of the RW impact on LR is conflicting. No randomised controlled trial (RCT) has been conducted, but a large SCRCR study, recent systematic reviews and meta-analyses have demonstrated a significant LR reduction when RW is performed [15–18, 21, 23]. An obstacle to performing an RCT is that power calculations indicate that a sample size of at least 1400 patients and a follow-up period of five years are needed [15]. Furthermore, during the establishment of the TME surgery most European colorectal surgeons adopted the technique and were convinced of the importance of RW. Therefore, some authors believe it would be unethical to perform an RCT [15, 16].

Our data come from a registry with high external and internal validity that contains all Swedish hospitals performing rectal cancer surgery and data that have been collected prospectively. In a validation of the SCRCR data, the validity of the variable RW was high [30]. The SCRCR data are truly population-based, unselected and reflect the average management of rectal cancer in Sweden. Thus, patients who would have been excluded in an RCT (e.g., due to age or comorbidity) are included in the analyses, rendering a large study population.

The Swedish national guidelines recommend RW be performed with sterile water or another cytotoxic solution when performing AR for rectal cancer [31]. The RW frequency in Sweden over the years has been approximately 90% in patients treated with AR according to the SCRCR [9]. This is in accordance with the findings in our cohort. Unfortunately, the reason for RW omission is not stated in the SCRCR. A survey of the current practice of RW in the UK showed that 87.2% of the responders performed RW in open resections, but only 54.8% of the responders who performed laparoscopic surgery routinely performed RW during laparoscopic resections for rectal cancer [26]. In our study, the proportion of laparoscopic procedures was higher in the no RW group. Meanwhile, a recent survey conducted by our group concerning the practice of RW in Sweden showed no differences on routine use of RW between open and minimally invasive surgery [32].

In a few case reports, adverse events after RW have been reported, such as anaphylaxis due to RW with chlorhexidine as well as blood pressure drop and cardiac ischemia after use of cetrimide [24, 25]. From our earlier study, we know that chlorhexidine alone and cetrimide are not used in Sweden [32]. In Sweden, sterile water or a mixture of sterile water and alcohol are the most common solutions followed by a mixture of either alcohol or chlorhexidine with sterile water or saline [32]. Despite the lack of evidence, many authors state that RW is safe and does not alter the risk of complications [15, 16, 19, 23]. This opinion is supported by our results but based on our earlier survey, this only applies to RW with sterile water as well as alcohol or chlorhexidine mixed with sterile water or saline, and not to other solutions [32].

The lack of randomisation between the groups is a limitation of our study. Furthermore, the SCRCR does not state how RW was executed, both in terms of solution and volume. The Swedish national guidelines for colorectal cancer care give no recommendation on the volume of fluid or on the technique to use [31]. In our recent study, we found that there are differences in practice among Swedish colorectal units on those two items [32]. A possible way to find answers concerning what RW solution and volume to use may be to include those variables in the SCRCR dataset. Furthermore, previous studies have shown that postoperative complications are underreported in the SCRCR [33]. However, there is no reason to believe that there is an uneven distribution of this underreporting between the groups in our study.

## Conclusions

Although RW might be a surrogate marker for overall quality of rectal surgery, our study suggests that RW with sterile water or an alcohol-based solution is a safe technique that does not increase postoperative complications. The routine to perform RW in AR with TME technique for rectal cancer in spite of the absence of RCTs is supported. Further work is needed to answer what technique, fluid and volume to use, so as to establish a consensus on these issues.

## Data Availability

The datasets used and analysed during the current study are available from the corresponding author on reasonable request.

## Abbreviations

AL: Anastomotic leakages
AR: Anterior resection
CHT: Chemotherapy
CI: Confidence interval
CRM: Circumferential resection margin
LR: Local recurrence
OR: Odds ratio
RCT: Randomised controlled trial
RT: Radiotherapy
RW: Rectal washout
SCRCR: Swedish Colorectal Cancer Registry
TME: Total mesorectal excision

## References

[1] Burton S, Brown G, Daniels IR, Norman AR, Mason B, Cunningham D (2006). MRI directed multidisciplinary team preoperative treatment strategy: the way to eliminate positive circumferential margins?. Br J Cancer..

[2] Erlandsson J, Holm T, Pettersson D, Berglund A, Cedermark B, Radu C (2017). Optimal fractionation of preoperative radiotherapy and timing to surgery for rectal cancer (Stockholm III): a multicentre, randomised, non-blinded, phase 3, non-inferiority trial. Lancet Oncol..

[3] Glimelius B (2012). Multidisciplinary treatment of patients with rectal cancer: development during the past decades and plans for the future. Ups J Med Sci..

[4] Heald RJ, Moran BJ, Ryall RD, Sexton R, MacFarlane JK (1998). Rectal cancer: the Basingstoke experience of total mesorectal excision, 1978-1997. Arch Surg..

[5] Kodeda K, Johansson R, Zar N, Birgisson H, Dahlberg M, Skullman S (2015). Time trends, improvements and national auditing of rectal cancer management over an 18-year period. Colorectal Dis..

[6] Peeters KC, Marijnen CA, Nagtegaal ID, Kranenbarg EK, Putter H, Wiggers T (2007). The TME trial after a median follow-up of 6 years: increased local control but no survival benefit in irradiated patients with resectable rectal carcinoma. Ann Surg..

[7] Keller DS, Berho M, Perez RO, Wexner SD, Chand M (2020). The multidisciplinary management of rectal cancer. Nat Rev Gastroenterol Hepatol..

[8] Glimelius B (2013). Neo-adjuvant radiotherapy in rectal cancer. World J Gastroenterol..

[9] Swedish Colorectal Cancer Registry. https://www.cancercentrum.se/samverkan/cancerdiagnoser/tjocktarm-andtarm-och-anal/tjock%2D%2Doch-andtarm/kvalitetsregister/. Accessed 21 Jan 2021.

[10] Fermor B, Umpleby HC, Lever JV, Symes MO, Williamson RC (1986). Proliferative and metastatic potential of exfoliated colorectal cancer cells. J Natl Cancer Inst..

[11] Gertsch P, Baer HU, Kraft R, Maddern GJ, Altermatt HJ (1992). Malignant cells are collected on circular staplers. Dis Colon Rectum..

[12] Umpleby HC, Fermor B, Symes MO, Williamson RC (1984). Viability of exfoliated colorectal carcinoma cells. Br J Surg..

[13] Okada K, Sadahiro S, Kamei Y, Chan LF, Ogimi T, Miyakita H (2020). A prospective clinical study assessing the presence of exfoliated cancer cells and rectal washout including tumors in patients who receive neoadjuvant chemoradiotherapy for rectal cancer. Surg Today..

[14] Dafnis G, Nordstrom M (2013). Evaluation of the presence of intraluminal cancer cells following rectal washout in rectal cancer surgery. Tech Coloproctol..

[15] Kodeda K, Holmberg E, Jorgren F, Nordgren S, Lindmark G (2010). Rectal washout and local recurrence of cancer after anterior resection. Br J Surg..

[16] Matsuda A, Kishi T, Musso G, Matsutani T, Yokoi K, Wang P (2013). The effect of intraoperative rectal washout on local recurrence after rectal cancer surgery: a meta-analysis. Ann Surg Oncol..

[17] Zhou C, Ren Y, Li J, Li X, He J, Liu P (2014). Systematic review and meta-analysis of rectal washout on risk of local recurrence for cancer. J Surg Res..

[18] Zhou C, Ren Y, Li J, Wang K, He J, Chen W (2014). Association between irrigation fluids, washout volumes and risk of local recurrence of anterior resection for rectal cancer: a meta-analysis of 427 cases and 492 controls. PLoS One..

[19] Okoshi K, Kono E, Tomizawa Y, Kinoshita K (2020). Can rectal washout reduce anastomotic recurrence after anterior resection for rectal cancer? A review of the literature. Surg Today..

[20] Moosvi SR, Manley K, Hernon J (2018). The effect of rectal washout on local recurrence following rectal cancer surgery. Ann R Coll Surg Engl..

[21] Siddiqi N, Abbas M, Iqbal Z, Farooq M, Conti J, Parvaiz A (2016). Benefit of rectal washout for anterior resection and left sided resections. Int J Surg..

[22] Jorgren F, Johansson R, Arnadottir H, Lindmark G (2017). The importance of rectal washout for the oncological outcome after Hartmann’s procedure for rectal cancer: analysis of population-based data from the Swedish Colorectal Cancer Registry. Tech Coloproctol..

[23] Rondelli F, Trastulli S, Cirocchi R, Avenia N, Mariani E, Sciannameo F (2012). Rectal washout and local recurrence in rectal resection for cancer: a meta-analysis. Colorectal Dis..

[24] Liu SY, Lee JF, Ng SS, Li JC, Yiu RY (2007). Rectal stump lavage: simple procedure resulting in life-threatening complication. Asian J Surg..

[25] Saeed WR, Stewart J, Benson EA (1991). Cetrimide lavage: ineffective and potentially toxic. Ann R Coll Surg Engl..

[26] Simillis C, Mistry K, Prabhudesai A (2013). Intraoperative rectal washout in rectal cancer surgery: a survey of current practice in the UK. Int J Surg..

[27] Danish colorectal cancer group. National annual report 2016. http://dccg.dk/wp-content/uploads/2017/10/Aarsrapport_2016.pdf. Accessed 1 Dec 2018.

[28] Pahlman L, Bohe M, Cedermark B, Dahlberg M, Lindmark G, Sjodahl R (2007). The Swedish rectal cancer registry. Br J Surg..

[29] Van Leersum NJ, Snijders HS, Henneman D, Kolfschoten NE, Gooiker GA, ten Berge MG (2013). The Dutch surgical colorectal audit. Eur J Surg Oncol..

[30] Moberger P, Skoldberg F, Birgisson H (2018). Evaluation of the Swedish Colorectal Cancer Registry: an overview of completeness, timeliness, comparability and validity. Acta Oncol..

[31] Regional Cancer Centres in Cooperation. Swedish National Guidelines for Colorectal Cancer. https://www.cancercentrum.se/samverkan/cancerdiagnoser/tjocktarm-andtarm-och-anal/tjock%2D%2Doch-andtarm/vardprogram/gallande-vardprogram/. Accessed 21 Jan 2021.

[32] Svensson Neufert R, Teurneau-Hermansson K, Lydrup M-L, Jörgren F, Buchwald P (2018). Rectal washout in rectal cancer surgery: a survey of Swedish practice. Int J Surg Open..

[33] Gunnarsson U, Seligsohn E, Jestin P, Pahlman L (2003). Registration and validity of surgical complications in colorectal cancer surgery. Br J Surg..

